# GIDL: a rule based expert system for GenBank Intelligent Data Loading into the Molecular Biodiversity database

**DOI:** 10.1186/1471-2105-13-S4-S4

**Published:** 2012-03-28

**Authors:** Paolo Pannarale, Domenico Catalano, Giorgio De Caro, Giorgio Grillo, Pietro Leo, Graziano Pappadà, Francesco Rubino, Gaetano Scioscia, Flavio Licciulli

**Affiliations:** 1 Institute for Biomedical Technologies (ITB), National Research Council (CNR), Bari, 70100, Italy; 2 Institute of Plant Genetics (IGV), National Research Council (CNR), Bari, 70100, Italy; 3 IBM GBS BAO Advanced Analytics Services and MBLab, Bari, 70100, Italy; 4 Exhicon srl, Trani, 70059, Italy

## Abstract

**Background:**

In the scientific biodiversity community, it is increasingly perceived the need to build a bridge between molecular and traditional biodiversity studies. We believe that the information technology could have a preeminent role in integrating the information generated by these studies with the large amount of molecular data we can find in bioinformatics public databases. This work is primarily aimed at building a bioinformatic infrastructure for the integration of public and private biodiversity data through the development of GIDL, an Intelligent Data Loader coupled with the Molecular Biodiversity Database. The system presented here organizes in an ontological way and locally stores the sequence and annotation data contained in the GenBank primary database.

**Methods:**

The GIDL architecture consists of a relational database and of an intelligent data loader software. The relational database schema is designed to manage biodiversity information (Molecular Biodiversity Database) and it is organized in four areas: MolecularData, Experiment, Collection and Taxonomy. The MolecularData area is inspired to an established standard in Generic Model Organism Databases, the Chado relational schema. The peculiarity of Chado, and also its strength, is the adoption of an ontological schema which makes use of the Sequence Ontology.

The Intelligent Data Loader (IDL) component of GIDL is an Extract, Transform and Load software able to parse data, to discover hidden information in the GenBank entries and to populate the Molecular Biodiversity Database. The IDL is composed by three main modules: the Parser, able to parse GenBank flat files; the Reasoner, which automatically builds CLIPS facts mapping the biological knowledge expressed by the Sequence Ontology; the DBFiller, which translates the CLIPS facts into ordered SQL statements used to populate the database. In GIDL Semantic Web technologies have been adopted due to their advantages in data representation, integration and processing.

**Results and conclusions:**

Entries coming from Virus (814,122), Plant (1,365,360) and Invertebrate (959,065) divisions of GenBank rel.180 have been loaded in the Molecular Biodiversity Database by GIDL. Our system, combining the Sequence Ontology and the Chado schema, allows a more powerful query expressiveness compared with the most commonly used sequence retrieval systems like Entrez or SRS.

## Background

We are living an historical moment characterized by deep changes in the life sciences due to the widespread use of new technologies. The huge production of molecular data obtained by high-throughput technologies are moving the research groups from "reductionist approach" to "holistic approach" studies.

For example, in the biodiversity domain, several researchers analyze molecular data obtained by high-throughput DNA sequencing technology and compare them with morphological and geographical data obtained by classical methods [1,2].

So new challenges arise in biology and the need for transforming large volumes of raw data into usable knowledge about our world and its inhabitants emerges. This transformation poses significant challenges that necessitate the assistance of automated methods. The organization of biological information from an array of resources into consolidated knowledge bases for subsequent archival and research purposes is a significant informatics task. Recent advances in information technology are creating a revolution in the way the biology data are organized, stored, integrated and distributed towards the development of testable hypotheses [3,4]. Several informatics efforts have been made to integrate molecular data stored in the National Center for Biotechnology Information (NCBI) [5] with other molecular public databases, or with public biodiversity data resources, as in the case of Global Biodiversity Information Facility (GBIF) [6], where the primary biodiversity data are correlated to the metadata and other information.

The centralization of heterogeneous data into a single resource is one of the possible solution as it can enable a range of comparative studies, can help the integration of locally produced data with bioinformatics public databases and can facilitate the computation of millions of data records carried out by modern bioinformatic software.

In the bioinformatics community, centralized systems like the Entrez system [7] at NCBI or SRS [8] at EBI provide access to biomedical information across many resources. Anyway, when researchers need a large amount of molecular data for their studies, public primary databases are an endless source of information, though difficult to harvest in case of large sets of sequences or entries. In fact, querying over the "net" has the disadvantage that large queries may take a long time to complete or may not return any results due to server or network resource restrictions.

To overcome this issue we have built an intelligent loading system (GIDL) that allows to extract, transform and store data in a target database schema, implementing a knowledge base constituted by public NCBI GenBank [9] entries including all entries from the International Nucleotide Sequence Database Collaboration-INSCD (DNA Data Bank of Japan-DDBJ [10], NCBI-GenBank and the European Molecular Biology Laboratory-EMBL [11] databases). Moreover, using Semantic Web technologies, the software is able to discover hidden information in the sequence entries. The combined use of semantics, ontology and information extracted from GenBank allows to perform more powerful query expressiveness in order to run advanced computational analyses.

Other similar applications are available in the bioinformatics community, like Biospida [12] or various data integration tools based on a data-warehouse approach, like Atlas [13] and BioWarehouse [14]. The advantage of our system is the use of a semantic approach not only to represent data extracted from the entries but also to infer and store new information useful to facilitate and increase the data retrieval capabilities.

The system described in this paper addresses some of the aims of the Molecular Biodiversity Laboratory (MBLab) project [15], a private-public initiative funded by the Italian MIUR (Ministry of Education, University and Research). Briefly, the project goal is the integration of biodiversity data coming from private collections or research activities with molecular data available in primary databases like GenBank.

## Methods

The overall GIDL architecture is composed by the **Target Database Schema**, dedicated to store ontology-driven information extracted from GenBank, and by the **Intelligent Data Loader (IDL)**, the core component which parses, adds semantics and populates the database. Most of the software implementation has been done in JAVA language, whereas we used Relational Database Management System (DBMS) to implement the Target Database Schema.

### Target database schema

A relational data model is used to represent all the information extracted from GIDL. The schema is designed considering all the biological entities involved in the molecular biodiversity domain. In this paper we refer to the Target Database Schema as Molecular Biodiversity Database schema. It encompasses four main areas: MolecularData, Experiment, Collection, Taxonomy. This schema is a part of a more extensive logical schema built by our group, in the MBLab project, to store and integrate public and private biodiversity data.

**Figure 1:**
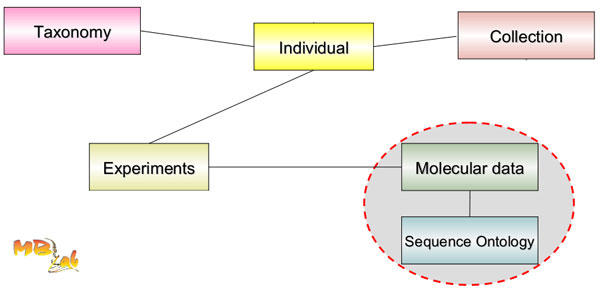
**High-level view of the database schema**. The relational schema is logically composed by four sections: MolecularData, Experiments, Collection and Taxonomy.

As depicted in Figure 1, the core entity of the schema is the "Individual", a material living entity. It is considered as a central element, linking all information and representing a biological entity characterized by several information (experiments, sequences, observations, etc.). The Individual is an entity that can be collected and stored in physical collections (e.g., a specimen in a museal collection, an accession of the seed in a genebank or a strain in a culture collection), where each instance of the Individual is labelled according to an official taxonomy. For example, an individual can be the source material for an experiment that can lead to morpho-phenotypic data, computational data, fragment profiles, chemical essays and sequencing data. A detailed representation of the relational schema is available at the MBLab project site [15].

The Molecular Biodiversity Database schema design is inspired by international standards used in the molecular biodiversity domain. The BOLD Barcode of Life Data System (BOLD) [16] schema has been used to represent taxonomic and collection data, while the MolecularData area is inspired to an established standard in Generic Model Organism Databases (GMOD) [17], the Chado [18] relational schema. The peculiarity of the Chado schema, and also its strength, is the use of an ontological schema. As ontological schema we mean a simple relational design in which entities and relationships have common relations. Individuals and entities, each one with different attributes and relationships, like genes, promoters, or repeated regions, are stored in the same table and labelled by the corresponding ontological terms. Also the relationships are stored in a common table, whereas attributes are stored in a separate table and linked to the entity table. The choice of this design is motivated by the nature of the biological domain, in which entities, attributes and relationships are really fickle, and such a rate of updates to the schema may be unsustainable or not convenient; but this comes at a price, the incapability to define constraints and to ensure consistency. These constrains are defined in the ontology, the formal representation of the knowledge domain, but implementing them in the relational environment goes beyond relational standard means. In fact, in a usual relational schema, each entity and relationship (e.g., gene, CDS, promoter and gene/CDS relationship) have a dedicated table. This design does not allow the definition of a promoter as part of a CDS, due to the constraints imposed by the schema. In our system, the schema-by itself-may allow the definition of a biologically incorrect relationship (e.g. CDS as part of a promoter), but GIDL, that populates the database instance, ensures data consistency granting only the relationships defined by the ontology.

In order to represent the semantic information related to biological sequences, Chado makes use of the Sequence Ontology (SO) [19], an accurate and continuous enrichment ontology belonging to the Open Biomedical Ontologies (OBO) consortium [20]. The SO was begun in 2003 as a means to provide terms and relationships to describe biological sequences; its main purpose is the unification of the vocabulary used in genomic annotations. SO provides a common set of terms and definitions that facilitate the exchange, analysis and management of genomic data. Because SO treats part-whole relationships rigorously, it can be used as a substrate for automated reasoning and instances of sequence features described by the SO can be subjected to a group of logical operations known as extensional mereology operators (EM) [21]. The SO was initially divided into aspects to describe the features of biological sequence and the attributes of these features. A sequence feature is a region or a boundary of a sequence that can be located through the coordinates on the biological sequence.

The proposed Molecular Biodiversity Database schema, according to Chado concepts, uses the same table for all different types of features, and uses the SO as a typing system, that is, it stores sequence features replaced by the corresponding SO terms. Sequence features info, SO terms and sequence features/SO terms mapping are stored in three different tables; the use of the latter guarantees that any future mapping change will not impact the sequence features and SO terms tables. Other sequence annotations (attributes) are stored in another table and mapped to SO too. In the schema implementation, each SO term is represented as a record in the OntologyTerm table. Another important table is the SequenceFeature table: its identifier constitutes a foreign key for tables storing feature annotations, feature evidences and relationships between annotations and evidences.

SO, through the *part_of *relationship, uses a subsumption hierarchy to describe the feature types and a meronomy (a type of hierarchy which deals with part-whole relationships) to describe their part-whole structures [19,21]. Features are related by their genomic position, e.g., polypeptides and transcripts are described by their genomic context.

The ontological organization of the data is made persistent in the CVPath table, that is part of the CHADO schema. It implements a transitive closure (pre-computed at setup time by an OWL reasoner) of the *is_a *relationship, thus allowing to retrieve items by a subsumption inference (e.g., when asking the database for all the features labelled as "genes", we will also retrieve the ones labelled as "mitochondrial genes").

The relational approach behind the design of the schema allows the use of any relational Database Management System (DBMS). In our prototype the Molecular Biodiversity Database schema is implemented in the IBM DB2 DBMS. So the query system performance of the prototype takes advantages of the DB2 performances due to the advanced indexing and storage capabilities.

### Intelligent data loader

GenBank sequence and feature data are populated in the Molecular Biodiversity Database by an Extract, Transformation and Loading (ETL) software we designed and implemented named Intelligent Data Loader (IDL). It is able to parse and add semantic information to the GenBank entries. IDL is composed by three main modules: the **Parser**, the **Reasoner **and the **DBFiller**.

#### Architecture

**Figure 2:**
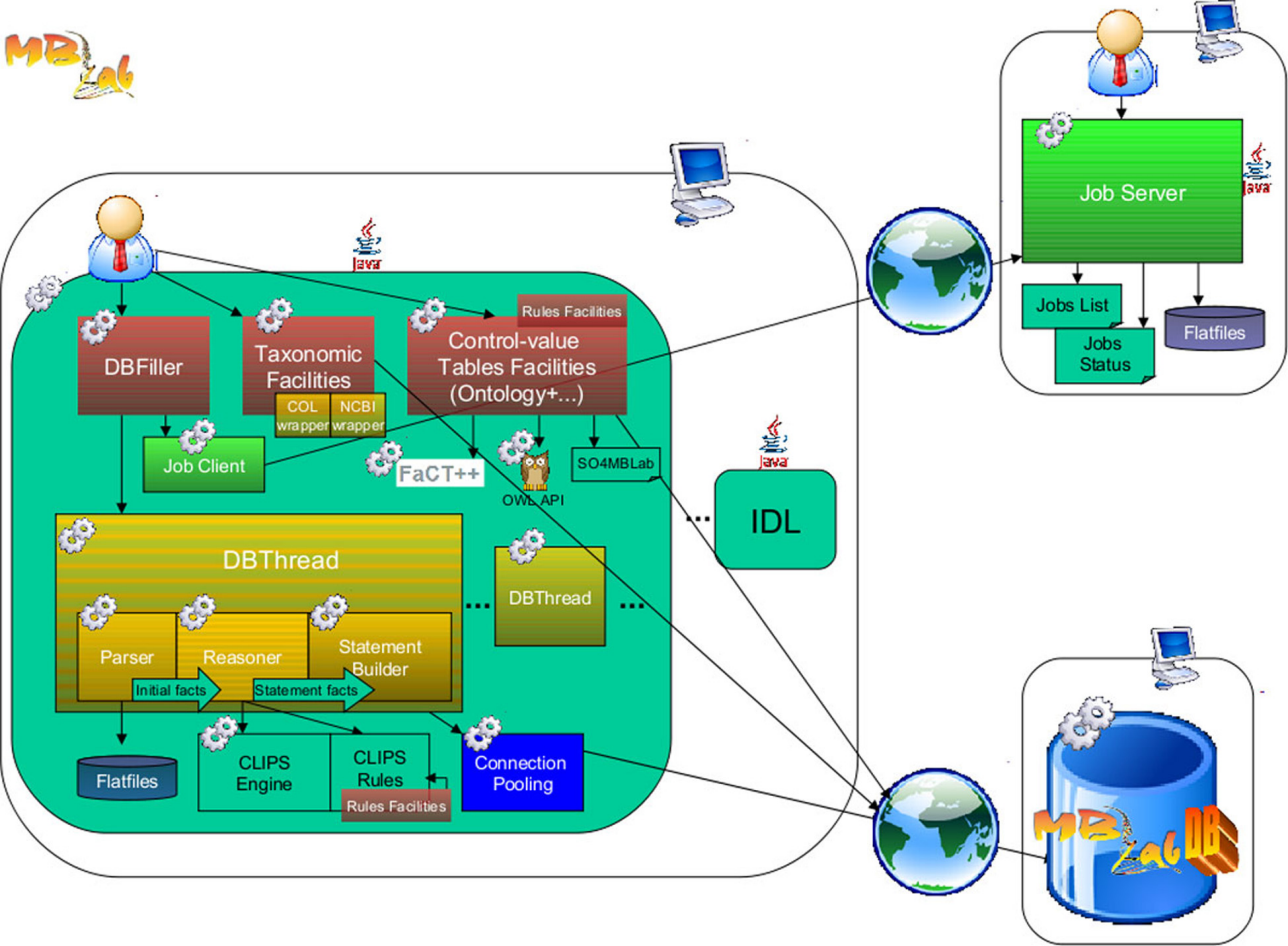
**Component Model of the GenBank data loader**. This process involves a number of components implementing a parallelized distributed system for a fast loading. We chose a Rule system that allows complex, scalable, maintainable conversion from the GenBank Feature Table Format to a Sequence Ontology based relational schema. The ontology allows, by an OWL reasoner, the automatic generation of the facts for the CLIPS component.

IDL architecture, as shown in Figure 2, is composed by three different applications: the data loader, the job server and a relational DBMS.

The job server indexes a local copy of the flat files to be loaded and draws up a list of entries, each one with an associated state: ready, pending, loaded. It performs the following operations: creates the list of jobs, returns a job to be executed, marks a job as completed, writes the list of completed jobs, re-adds jobs dispatched but not completed to the "to-do" list.

The creation of the job list consists of a set of records (jobs or entries) each one keeping the information about the seek position of the entry first line in the flat file (each flat file contains many entries), the seek position of the first nucleotide of the sequence and the current GenBank GI number of the entry.

When the client asks for a new job, some pieces of data are sent to it via socket communication and the job is set in the pending state. When the DBThread component finishes to process the entry, it sends a "completed" message to the server and the job can be set in the completed state. The DBFiller, once in a while sends a message to the server to write in a persistent way the status of the jobs in the list. If a job runs up against an exception during its processing, remaining in the pending condition, it can be processed again after a reset of the pending jobs list. By means of the job server, IDL handles faults and exceptions and implements the upgrading procedure necessary to load new entries available in a new GenBank release.

The IDL, through an OWL reasoner, automatically builds CLIPS [22] facts reflecting biological knowledge expressed in the Sequence Ontology at setup time. These facts are afterwards used by the DBFiller module, that translates them into ordered SQL statements which are used to populate the schema.

The IDL, the job server and the DBMS can be scattered on different machines in order to distribute the computational load, as we discuss in the following.

#### Parser

Currently, several parser libraries for the GenBank flat-files have been developed, such as BioJava [23], BioPython [24], BioPerl [25], the AJAX library in the EMBOSS package [26] and the C library GBParsy [27]. All of these are general purpose libraries that populate in-memory data structures, reflecting the parsed flat file. This type of generality comes at the price of reduced performance. The BioJava package was initially employed in GIDL; but, after having noticed it being a substantial bottleneck, a mixed regular expression and content-specified functions based parser was developed in-house. The parser was designed following the specifications of the DDBJ/EMBL/GenBank Feature Table definition FTv8 [28].

Initially, four portions of the entry are localized within the GenBank flat-file: the header, the references, the features and the sequence section. The nucleotide sequences are not translated into a clips fact in order not to overload the reasoner. Some regular expressions are applied to extract the information contained in the header string, then the header is translated into a single clips header fact. The references string is split into several single reference strings, the regular expressions are applied and each reference is translated into a reference fact with one or more reference location, i.e. the features a reference is the evidence of. The features are initially indexed to locate each feature string within the file; those indexes are then passed to a method that builds a feature object. Each feature object owns one or more locations and qualifier objects, extracted by means of regular expressions. Finally each feature object is serialized into a key fact in relationship with several location and qualifier facts.

#### Reasoner

IDL makes use of two different reasoners: an OWL Reasoner (Fact++) and a CLIPS Reasoner.

The OWL [29] reasoner acts at a preliminary stage, *una tantum*, in the setup phase of the IDL execution. It is used to make all the admissible relationships among features explicit and to consequently build CLIPS facts representing the knowledge in the Sequence Ontology.

In this phase a Java program, given an SO term and an SO object property, creates an OWLObjectPropertySomeRestriction [30] proposition and asks the OWL reasoner for all the descendant classes that match the restriction. The process is repeated for all the Class-Object property combinations and the resulting triples are added to the Clips knowledge base as initial facts. The procedure is executed only once at setup time or each time the sequence ontology changes, so the IDL loading performances are not affected by this reasoner.

On the other hand, the CLIPS reasoner is the component dedicated to the ETL rules execution. This reasoning environment is based on CLIPS, one of the most popular software tools for building expert systems (further details in [22]). It makes use in turn of facts automatically generated by the previous OWL reasoning. These facts are used, at runtime, by the encoded CLIPS rules to find the relationships between features, using criteria based on qualifier values. Only the admissible relationships survive to the evaluations of the reasoner and are inserted in the database. A working example of the GIDL execution pipeline is provided in the Additional File 1.

In our implementation, a representation based on one header and one sequence, and one or more key, portion, reference and reference_portion templates are transformed into an ordered set of statements in the form of insert and select templates. The entry-templates have all the slots required by the DDBJ/EMBL/GenBank FTv8.

**Figure 3:**
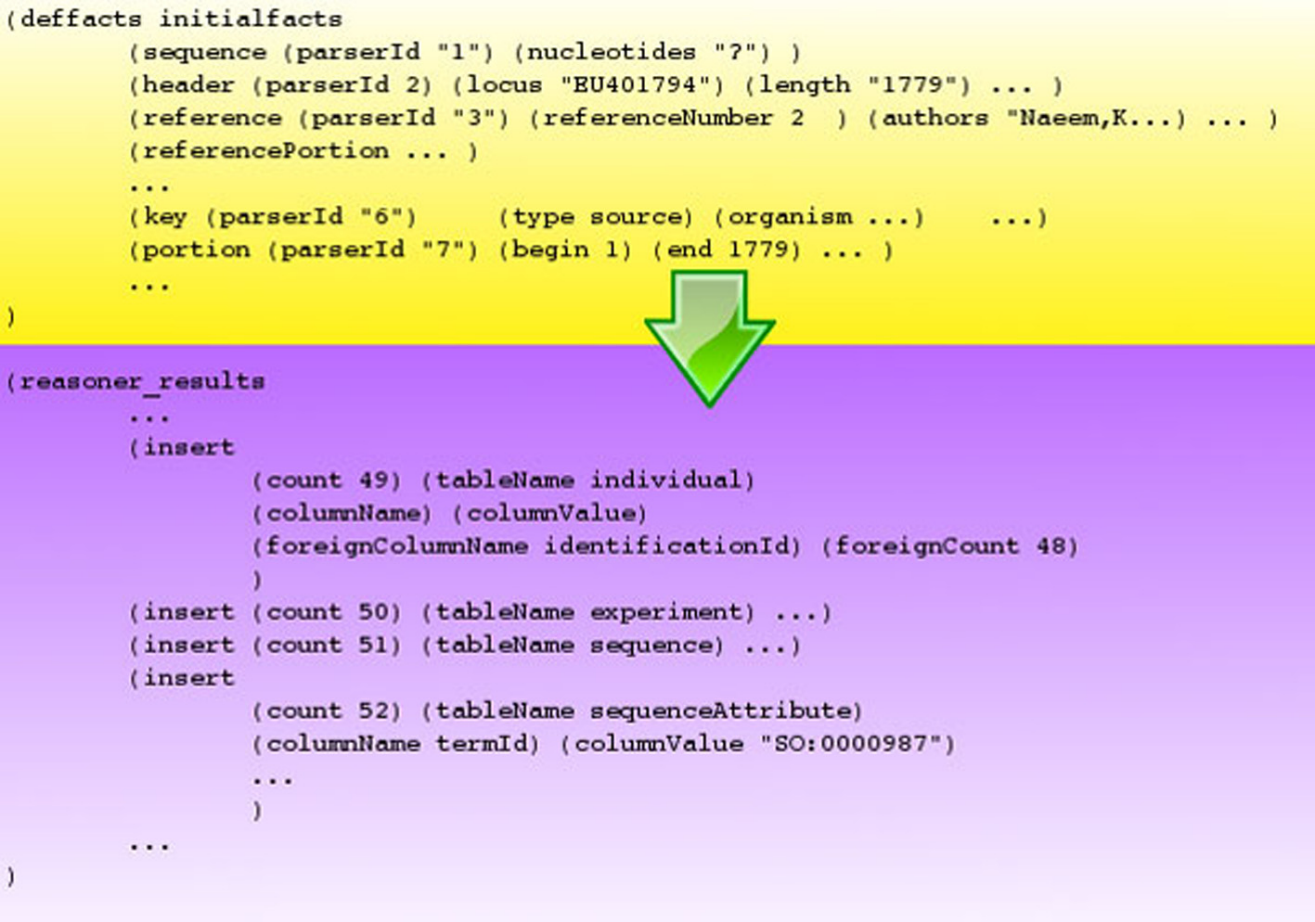
**Flat file and statement templates**. CLIPS templates define the form that facts can take. Every time a fact of this type is created, it contains the slots specified in its definition, each of which can contain a value and can be accessed by name. We use the header, sequence, key, portion, reference and reference_portion templates for flat files and insert and select templates for statements. The rule engine performs the conversion.

The insert template includes a "tableName" slot to identify the involved table and two multislots, "columnName" and "columnValue", to identify respectively the columns and the values. Two more multislots have been conceived to manage the cross-reference of foreign keys that refer to automatically generated keys. Each statement is identified by a "count" slot; this identifier is used in the "foreignCount" multislot of a different insert to refer to the first one. This value will be substituted in run-time with the real value assigned by the DBMS. As an example, in Figure 3, in the 49th statement, the value of the column "identificationId" will be substituted in run-time by the value assigned by the DBMS to the primary key of the record generated by the insert 48.

During the transformation process each feature key type was mapped to an SO term using the correspondence given in the feature table SO mapping file [31], made publically available and updated by the Sequence Ontology authors. Some keys in the mapping were missing but the corresponding concepts were found in the SO and mapped in the rules, whereas for some sequence or feature attributes that were not present in the SO, either the GenBank qualifier or the header field name were used.

Since bibliographic references in the FTv8 are assigned to specific sequence locations, a relationship between the feature and its bibliographic evidence was assigned basing it on location coherence.

The Rule Facility also produces rules for the translation of qualifiers into SQL statements for those qualifiers that didn't need to be loaded into the Collection portion of the schema. These rules are not the result of an OWL reasoning. Instead, biodiversity specific qualifiers, including experimental, taxonomic and sampling details, were managed through rules coded with the help of a domain expert, and stored in specific tables of the database (e.g. the Collection area). Generally speaking, whenever any qualifier had to be elaborated and stored in dedicated tables other than the Chado schema tables, a specific rule had to be coded. The other pieces of information are managed by the näive rules generated by the Rule Facility.

Finally, the *member_of*, *part_of *and *derives_from *relationships among features of an entry were reconstructed. The subsumption and transitive closure entailments were used to compute all the biologically possible relationships among types of features. This information was derived by the SO, and the corresponding knowledge was made available in CLIPS by the Rule Facility, as previously described. Feature-specific relationships were assessed on the bases of the feature types and of the qualifier and location consistency. The qualifiers used were *locus_tag*, *gene*, *product *and *protein_id*. The latter is an example of implicit information that can be easily extracted from the GenBank entries, coding appropriate rules.

#### DBFiller

**Figure 4:**
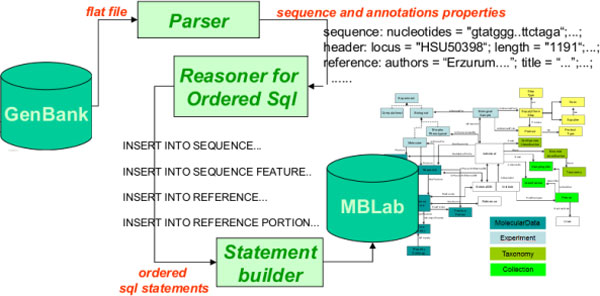
**Loading tasks**. In order to load the GenBank data in our relational sequence ontology based schema, we perform a complex conversion. This process starts by parsing the GenBank entry and transforming it into a set of facts understandable by a rule system based on the CLIPS rule engine. A set of rules, encoded in the reasoner, transforms the parser facts into statement facts, that correspond to an ordered list of insert and select statements addressed to a relational DBMS instance.

The DBFiller component asks the job client for a set of ready-state entries and assigns each one to a DBThread (an extension of ours for a Java Thread). The threads can be run with a configurable degree of parallelism to maximize loading performances. Each DBThread performs three steps: parsing, reasoning and statement building. The parser transforms a flat file in a set of CLIPS facts, that is, the initial facts. The reasoning component performs a call to the CLIPS engine through a Java Native Interface. The initial facts combined with the rules, partially encoded by a human expert and partially derived from the ontology, give rise to the reasoner results, i.e., a set of statement-like facts. The DBThread extracts the statement facts and builds real SQL statements that can therefore be submitted to the DBMS. After the execution of a configurable set of jobs, the DBFiller commits the job handling results to the Job Server, that is the main controller class in the IDL architecture. The pipeline is summarized in Figure 4.

Connections to the DBMS are managed by means of a connection pooling that allows a considerable time saving: the connections don't need to be opened and closed by each thread, but they are shared-in turn-among threads.

### System deployment topologies

**Figure 5:**
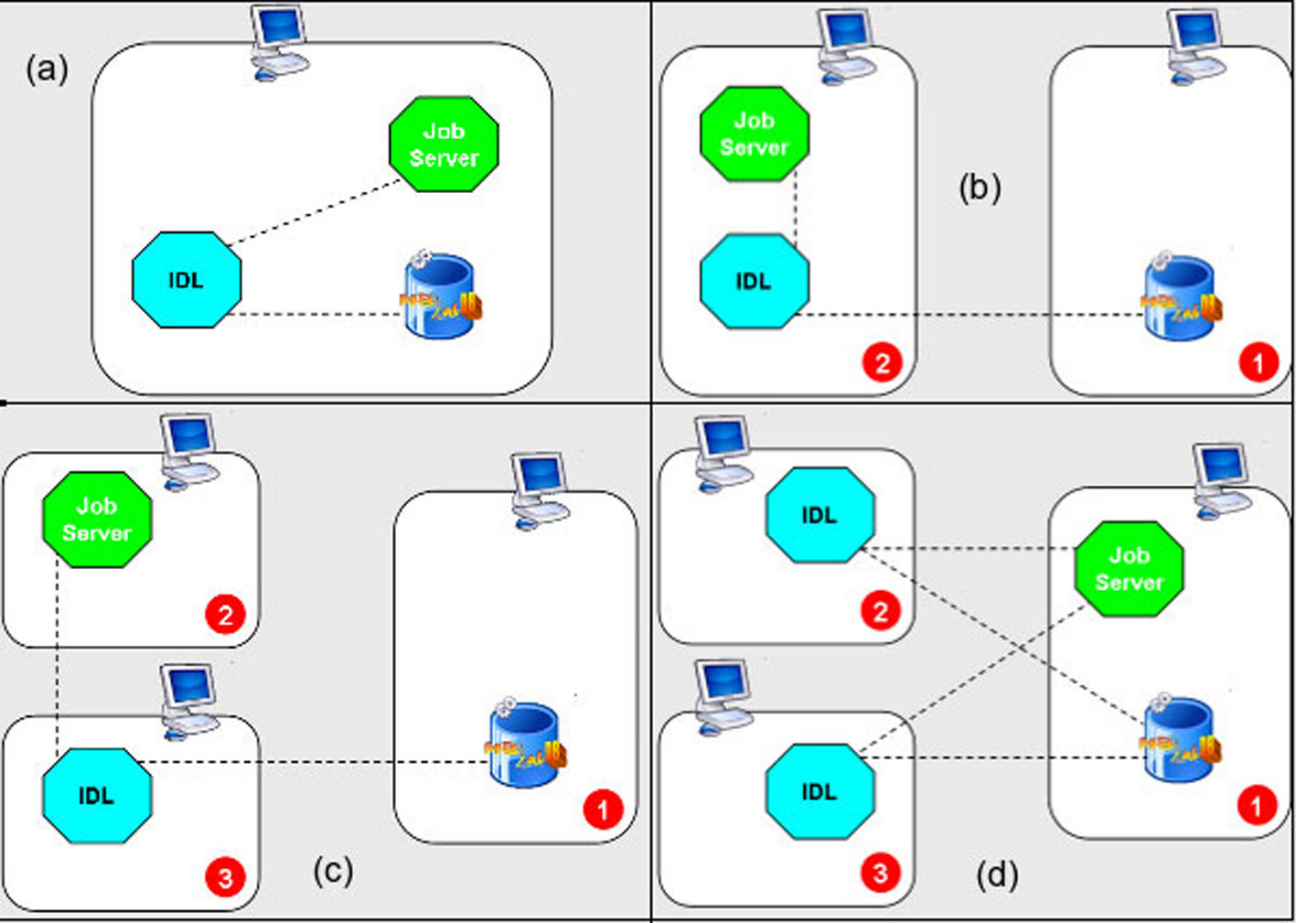
**Data Loader deployment topologies**. Some of the different topologies that the modular architecture of the Data Loader system allows. Case (a) refers to the simplest deployment, with all components in a box; cases (b), (c) and (d) refer to configurations in which the Data Loader components are deployed on two or more servers.

The modular solution conceived for the IDL allows the system to be deployed in different topologies, involving a different number of servers. The Figure 5 just shows some possible deployment topologies. In this figure the case (a) is the most compact solution, where all components are deployed in a single box (all-in-one). Such a solution could be suitable for small installations, where the number of entries to be loaded is quite small (less than a million), and refers to short sequences with relatively few annotations (not entire genomes!): in this case neither the DBMS nor the IDL will require too many resources in terms of CPU and RAM. A typical topology, suggested for most of the cases, is that shown in Figure 5(b), where the DBMS has been decoupled by the application components (Job Server and IDL). This solution proposes the best balance between the two resource-expensive components: the DBMS and the IDL instance. Figure 5(c) is just a showcase for a completely scattered configuration, where the three components are distributed on three separate boxes. It is not useful for practical cases, since the Job Server Process is quite inexpensive, from the CPU point of view, and is characterized by a very small memory footprint (less than 1 GB). Finally, Figure 5(d) depicts the most aggressive case, suitable for loading procedures that involve a large number of entries (several millions) with huge sequences (entire genomes) annotated with a lot of features. In this case, when two (or more) IDL instances work in parallel on different boxes, a fast network connection among the servers is desirable (in order to rapidly satisfy the large number of queries the IDLs will send against the DBMS), as well as a lot of RAM (8 GB or more) both on the IDL boxes and on the DBMS server.

## Results

GIDL was used to parse and load in the Molecular Biodiversity Database the entries extracted from four GenBank divisions (rel.180): VRL, INV, PNL and EST. VRL is the set of entries of the complete Viral division (814,122 entries); INV refers to the set of entries of the complete Invertebrate division (959,065 entries); PLN concerns the entries of the complete Plant division (1,365,360 entries); finally, we denote with EST a sub-set of 1,035,087 entries of the GenBank division of Expressed Sequence Tag belonging to organisms of the family *Asteraceae*. The GIDL tool arranged the entries in 50,758,000 sequence feature annotations and associated 13,298,000 instances to 1,342 Sequence Ontology terms.

We used GIDL to support a set of bioinformatics applications developed in the Molecular Biodiversity Laboratory (MBLab) [15]. Firstly, the GenBank data extracted and organized by GIDL was integrated with private biodiversity collections to help the MBLab researchers to exploit new knowledge in the molecular biodiversity domain. Other MBLab applications, like "Barcode Primers Discovery & Retrieval" [15] and "miRNA Target Comparison Tool" [15] have benefited from the GIDL implementation.

### Performance analysis

Loading performances are crucial in a data loading system that manages huge volumes of data as those involved in the bioinformatics domains. In order to evaluate the GIDL performances, this component has been armed with a logging sub-module that traces relevant loading parameters in an appropriate table of the database instance it works on. Our loading test has been performed with the four sets of entries previously described. Some of the most relevant observable parameters traced by the logging sub-module of GIDL are synthesized, in a synoptic view in Table 1, where rows refer to eight parameters for each entry-set, while columns show respectively, minimum, maximum, average and standard deviation values for each parameter observable within the corresponding set. As we can infer from the last column, the four sets are roughly equivalent in terms of cardinality (number of contained entries). For each set, the first four rows contain data for observables strictly depending on the set itself, such as the length of the DNA sequence (*SequenceLength*) in base pairs; the length of the feature table of the GenBank entries (*FatureTableLength*), measured in bytes; the number of features in the entries (*# Features*); and the number of SQL INSERT statements (*# Statements*) produced by the IDL, in order to insert each entry in the relational database in terms of the database schema discussed above. All these parameters depend on the sets, and neither on the computational capabilities of the server used nor on the topology chosen in the deployment phase.

**Table 1:** Synoptic report of the loading procedure

DIVISION		MIN	MAX	AVERAGE	Standard Deviation	# ENTRIES
**VRL**	**SequenceLength (bp)**	9	1181404	1097	4206	814122
	**FeatureTableLength (byte)**	716	887303	2504	3556	
	**# Features**	2	2894	4.5	8.8	
	**# Statements**	22	27367	52.1	94.5	
	**ParsingTime (ms)**	1	1925	6.5	10.0	
	**ReasoningTime (ms)**	72	173912	95.6	235.3	
	**InsertTime (ms)**	361	531333	759.3	6472.2	
	**TotalTime (ms)**	446	531467	861.5	6491.5	

**INV**	**SequenceLength (bp)**	7	3291871	1536	17715	959065
	**FeatureTableLength (byte)**	710	1536275	2222	6264	
	**# Features**	2	4033	4.5	22.0	
	**# Statements**	24	42815	51.9	197.9	
	**ParsingTime (ms)**	1	11044	6.1	24.4	
	**ReasoningTime (ms)**	67	391901	101.6	1020.6	
	**InsertTime (ms)**	503	1702407	670.2	4689.0	
	**TotalTime (ms)**	573	2098246	778.0	5406.3	

**PLN**	**SequenceLength (bp)**	2	3439086	2378	16960	1365360
	**FeatureTableLength (byte)**	821	1844292	2397	7106	
	**# Features**	2	4113	4.6	17.5	
	**# Statements**	23	33032	51.4	185.8	
	**ParsingTime (ms)**	1	5546	6.3	17.4	
	**ReasoningTime (ms)**	73	5803323	107.5	5633.7	
	**InsertTime (ms)**	423	401933	559.0	1518.5	
	**TotalTime (ms)**	501	6206793	672.8	6315.6	

**EST**	**SequenceLength (bp)**	7	1770	659	193	1035087
	**FeatureTableLength (byte)**	1358	4798	2563	351	
	**# Features**	2	2	2	0	
	**# Statements**	25	34	30.1	1.1	
	**ParsingTime (ms)**	3	233	6.1	2.8	
	**ReasoningTime (ms)**	73	1089	83.3	10.9	
	**InsertTime (ms)**	372	38746	499	959.1	
	**TotalTime (ms)**	454	38859	588.4	950.6	

This table shows, in a synoptic view, the main parameters of the loading procedure to populate the Molecular Biodiversity Database. These numbers refer to entries coming from four GenBank divisions (VRL, INV, PLN and EST). For each of these sets, the first four rows describe some aspects of the GenBank entries, while the other four ones refer to parameters measured during the loading procedure. The VRL and INV sets were loaded by using the topology shown in Figure 5(a), while the other sets were loaded by using the topology shown in Figure 5(b). See text for a complete discussion of this topic.

On the other hand, the following four rows for each data set refer to measured times that are, in principle, dependent on the computational setup. They refer, respectively, to the time spent to parse the entry (*ParsingTime*, measured in milliseconds), the elaboration time taken by the Reasoner (*ReasoningTime*), the time needed by the DBFiller to send all the composed SQL INSERT statements against the database (*InsertTime*). Finally, the total time to elaborate each entry is reported (*TotalTime = ParsingTime + ReasoningTime + InsertTime*).

In order to evaluate the influence that different topologies can imply in the loading performances, we carried out the loading of VRL and INV sets with a configuration as that depicted in Figure 5(a), while we used the Figure 5(b) topology to load PLN and EST sets. All boxes are servers 2 × Genuine Intel XEON quad core 2.66 GHz, 8 GB RAM, 20 GB swap disk connected in an internal network at 1 Gbps, equipped with Red Hat Enterprise Linux 5.1. Note that swap space is especially important for the IDL processes when large entries (with thousand of features) are elaborated: in these cases each IDL thread can use up to 10 GB or more of RAM/swap memory. In our tests the IDL was configured to run with two parallel threads, that is two threads running in a single Java Virtual Machine (JVM).

We can gather one of the most interesting aspects of the influence of the different topologies by focusing on the two sets INV and PLN. They are structurally very similar, since the average number of features per entry is 4.5 and 4.6 respectively, and the average number of produced statements per entry is 51.9 and 51.4. In this case, switching from topology (a) to topology (b) leads to an improvement of about 15% in the TotalTime (672.8 ms instead of 778 ms), and this is principally due to the improved InsertTime (559 ms against 670.2 ms, i.e. ~ +20%). This means that in the "INSERT phase", when IDL sends INSERT statements and the DBMS executes them, a configuration with these two components on different boxes is preferable. The gain due to more resources for computation is higher than the loss due to network latency. This and other considerations, however, indicate topology (b) as the preferable configuration with respect to any others.

### Testing procedure

A scrupulous testing procedure was needed to verify the correctness of the CLIPS rules and the correct insertion of the GenBank entries into the MBLab Database. In order to accomplish this task, a "controlled dataset", i.e. a relatively small dataset containing entries with pre-established characteristics, was prepared.

We selected five GenBank divisions (BCT, INV, PLN, VRL, VRT) and we scanned all the entries for each division to find and extract a subsample containing at least two entries for each of the 60 GenBank feature keys. Thus we obtained 492 entries, instead of the expected 600 (5 division × 60 feature keys × 2 sample entries) due to the lack of the selected feature key in some entries (a feature key may or may not be present in a particular division). Following the same procedure for the 96 available GenBank qualifiers, we extracted 824 entries, instead of the expected 930 (as for the feature keys, some qualifiers are not applicable to certain GenBank divisions). So the resulting controlled dataset included a total of 1316 entries. Using the IDL those entries were loaded in a Target Database instance dedicated to the test.

**Figure 6:**
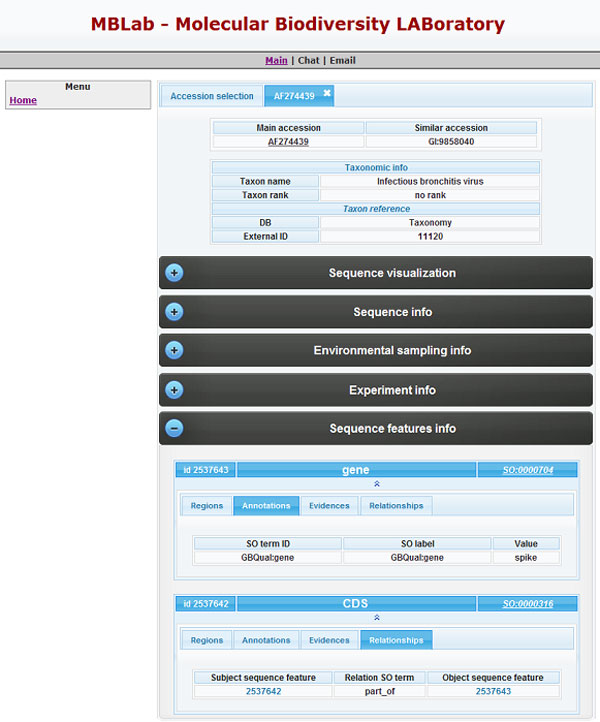
**GenBank entry consultation**. After selecting one or more accessions, it is possible to visualize the entries. The taxonomic data are immediately available. Other entry information are structured in different boxes, so a user can view these info only if a specific requests is made. We point out to a feature box: a distinctive view is implemented in the relationship tab; thanks to GIDL it is possible to see entry features related to a given feature.

The entries were parcelled out and delivered to a panel of 20 bioinformatics researchers with knowledge of the GenBank entry structure. The researchers received the entries to be checked in flat file format, a spreadsheet file, and the access to a minimal web interface of the database (Figure 6). For each assigned entry the testers had to mark, in the spreadsheet file, errors or discrepancies between the content of the entry in the flat file and the entry information loaded in the database. In order to check the reliability of the tests, some entries were assigned contemporarily to two or more testers.

After the reports were gathered and the detected bugs corrected, a second test session was performed using the same dataset. In this session 5 entries per person, different from those assigned before, were assigned to be checked with the same procedure for both header and feature key sections. This final session of tests ensured that the code debugging was carried out extensively and correctly.

### Query system prototype

By means of a JDBC SQL client access it is possible to query and retrieve data stored in the Molecular Biodiversity Database, populated by the Intelligent Data Loader. As a demonstration of GIDL capabilities we have built a graphical web interface querying prototype on top of the Molecular Biodiversity Database. The interface was used by trained bioinformatic researchers to test the correctness of IDL (illustrated in the Testing Procedure). The web interface prototype implements the use case of structured consultation of a GenBank entry. Entry information (sequence visualization, sequence info, environmental sampling info, experiment info and features info) are shown in separate dynamic boxes and are exploitable on request; so using this web interface the entry consultation is more concise than the GenBank website visualization.

Information about the features are further structured and for each of them it is possible to explore portions, nucleotides, annotations, evidences and relationships (according to the SO) with other features (see Figure 6).

Thanks to the ontology-driven schema implemented in GIDL, it is possible to query the Molecular Biodiversity Database taking advantage of logic relationships among features implied in GenBank entries and structured by the SO. To test this functionality the first version of the query system prototype has been enhanced with the ability to execute ontological queries.

**Figure 7:**
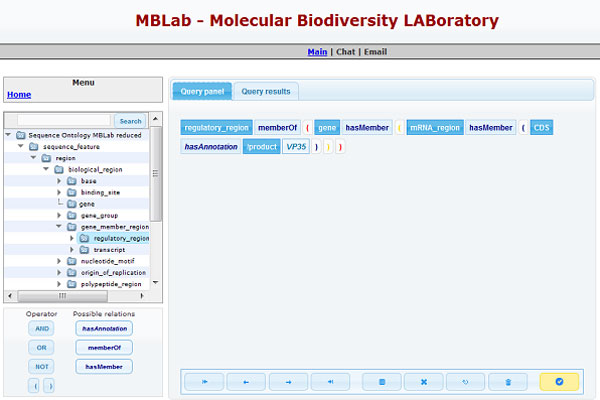
**Ontological query composition**. Using a tree view it is possible to build a query defining the relationships across the SO terms by memberOf (ancestors) or hasMember (descendants) relationships. To complete the query, the user has to define a particular annotation for the last SO term, selecting the annotation type (GenBank feature qualifier) and the correspondent annotation value. This figure shows the retrieval of all regulatory regions belonging to a gene, including the mRNA region which contains the CDS with '*VP35' *as annotation of the product value.

As an example, the user can make a query asking for regulatory regions (Sequence Ontology term: 'regulatory_region') of genes having a coding sequence (Sequence Ontology term: 'CDS') with a particular defined annotation. In our query system prototype, it is possible to formulate this query by means of a tree view (see Figure 7):

$$\begin{array}{c} \;\;\;\;regulatory\text{\_}region\;member\;Of(gene\;has\;Member\\ \left(CDS\;has\;Annonation<annotationType><annotationValue>\right)) \end{array}$$

where *< annotationType >*is a GenBank feature qualifier belonging to the CDS feature key, and *< annotationValue >*is the value for the CDS feature qualifier annotation.

Query results will consist of all entries satisfying this criterion. In this way it is possible to retrieve features (regulatory regions as in the above example) defining a criterion not only on that particular feature, but also on features related to it. Moreover query results will provide not only the features mapped to the SO term '*regulatory_region' *but also the features mapped as '*regulatory_region' *descendants (i.e., *polyA_signal_sequence*, *TF_binding_site*, *terminator*, ...) according to the '*is_a' *SO relations.

We want to point out that the web interface is just a prototype, with reduced functionality which does not allow currently to explore all the features of the Molecular Biodiversity Database. Further developments are in progress and out of the scope of this paper.

## Discussion

Frequently, in the molecular biodiversity domain in order to accomplish properly their analyses, the researchers need to retrieve large quantity of data (typically sequences and annotations) from remote public databases, store them locally and integrate these information with data contained in private databases and collections. To carry out this task, researchers may usually spend about 80% of their time in retrieving, assembling and preparing data for analysis (e.g. manipulating data, extracting subsets of data from external files, reformatting data, moving data, etc.) [32].

Our aim was to develop an intelligent solution to parse, retrieve and load the entire GenBank database locally. In fact, giving scientists access to a system in a high-bandwidth local network enhances large queries response and the data could be easily retrieved with lower latency than attempting to retrieve the same data over the Internet, like "net" solution (e.g. bioinformatic web-services, NCBI-Entrez and EBI-SRS sequence retrieval systems, etc.) [33].

Furthermore, we believe that GIDL, by means of the semantic web approach, has at least two advantages comparing it with other similar tools. First of all, it provides the users with an innovative way to query molecular data annotations. The integration of the SO knowledge base in GIDL allows to execute complex biological queries in a single step (as in the example illustrated in Figure 7). The same results are obtained by SRS and Entrez in several steps using multiple queries. Hence, the use of GIDL increases end-users productivity decreasing the number of steps in query building and execution.

Using all of the relationships in SO allows us to automatically draw logical conclusions about data that have been labelled with SO terms, and thereby provides useful insights into the underlying annotations [21]. GIDL is able to extract non explicit biological information from flat file entries and to add this new knowledge to the Target Database Schema. In many cases, a specific information which is critical for a given research is available but hidden (not explicit) in the GenBank flat files. For example, the feature key *intron *is not present in all entries, but GIDL allows to compute and store in the database, where conceptually feasible, the introns locations and sequences. In GIDL, just adding a simple new "biological" rule to the GIDL knowledge-base can give an off-the-shelf solution to data needs.

Despite loading and managing large primary biological databases (such as GenBank) in local DBMS is computational intensive, GIDL overcomes these obstacles satisfying the following requirements:

- **flexibility**: the user can easily re-define the extraction procedure logics and adapt it to his needs. The system would be able to adapt to changes in the knowledge of the biological domain. Actually, compared to traditional programming techniques, expert-system approaches provide more flexibility (and hence an easier modifiability) modelling rules as data rather than as code. The flexibility of the rule based approach also allows to easily manage the changes of the database schema or of the ontology. Although a rule based system can be perceived as more complex than an object-oriented system, and the object structure as more useful than the rule modularization and documentation, an initial steeper learning curve can give an effort ease later on;

- **speed**: the IDL application parses and transforms millions of GenBank entries and generates and executes tens of millions of SQL statements in a reasonable time;

- **knowledge representation**: the relationships between the entities of the entry and the attributes of those entities, give rise to implicit knowledge that has to be made explicit according to knowledge of the domain, as discussed before;

- **updatability**: the loader is able to manage frequent updates of new GenBank release;

- **robustness**: the modular design conceived for the IDL system (see Figure 2) makes it very reliable and flexible. In fact, as discussed before, the process named Job Server is the solely responsible for the management of the loading procedure. By an appropriate usage of an internal loading index, cached on a persistent support (file system), it is able to guarantee data integrity and fault tolerance. This means that if either the Job Server or any IDL instance failed, the entire loading procedure would not be compromised as a whole, since it would be sufficient to restart the failed process from the point of failure;

- **scalability**: another interesting feature of this data loading system is its (horizontal) scalability. In the "System deployment topologies" section we thoroughly discussed the numerous possibilities of deployment topologies its architecture makes possible. These solutions can cover scenarios ranging from the loading of small data sets up to very large databases in a reasonable time.

## Conclusions

In this work we have presented the concepts, the design and the implementation of the GIDL system, a toolkit consisting of a relational DB schema (based on the Molecular Biodiversity Database) and of an Intelligent Data Loader software that parse GenBank public entries, add semantics and load these information into an ontology-driven schema. The toolkit can be downloaded on request and installed by users who want to use a local instance of GIDL. Future developments for GIDL include the development of software modules to provide programmatic accesses via Application Programming Interface (API) in order to perform useful automatized operations on biological data.

## List of abbreviations used

BCT: Bacterial GenBank division; DB: Database; DBMS: Database Management System; GIDL: GenBank Intelligent Data Loader; EST: Expressed Sequence Tag GenBank division; ETL: Extraction, Transformation, Loading; FTv8: DDBJ/GenBank/EMBL Feature Table definition ver.8; IDL: Intelligent Data Loader; NCBI: National Center for Biotechnology Information; PLN: Plant GenBank division; SO: Sequence Ontology; VRL: Viral GenBank division; VRT: Vertebrate GenBank division.

## Competing interests

The authors declare that they have no competing interests.

## Authors' contributions

PP designed and developed the Molecular Biodiversity Database schema, the CLIPS rules and drafted the manuscript; DC participated to the schema design and drafted the manuscript; FR implemented the feature table to SO term mapping; GDC developed the query prototype interface and participated to the schema development: GG participated to the feature table to SO term mapping implementation and participated to the schema development; PL participated to the architectural design of the system and coordinated the IBM team; GP carried out the testing procedure and participated to the development of the parser for the GenBank entries; GS conceived the study, designed the system architecture, participated to the schema design and drafted the manuscript; FL conceived of the study, participated to the schema design, drafted the manuscript and coordinated the work. All authors read and approved the manuscript.

## Supplementary Material

Additional file 1 **PDF file containing a working example of the GIDL execution pipeline**. Click here for file
