# Supplementary material for: GIDL: a rule based expert system for GenBank Intelligent Data Loading into the Molecular Biodiversity database
Source: BMC Bioinformatics. 2012 Mar 28;13(Suppl 4):S4. doi: 10.1186/1471-2105-13-S4-S4 (PMC3303717; doi:10.1186/1471-2105-13-S4-S4)
Supplement: Additional file 1 — PDF file containing a working example of the GIDL execution pipeline. [file 1471-2105-13-S4-S4-S1.pdf]

Additional file 1

Example of GIDL execution pipeline

GIDL execution pipeline

This document reports a working example of the GIDL execution pipeline based on the GenBank entry AM744977. In the following paragraphs, in order to make easier the understanding of the data transformation process, data related to the same section of the GenBank entry (i.e., 'gene' and 'CDS' feature keys) has been boxed.

GenBank Entry

```
LOCUS       AM744977                3942 bp    RNA     linear   VRL 06-OCT-2009
DEFINITION  Epizootic hemorrhagic disease virus (serotype 1 / strain New
            Jersey) segment 1.
ACCESSION   AM744977
VERSION     AM744977.1  GI:260161523
KEYWORDS    complete genome segment.
SOURCE      Epizootic hemorrhagic disease virus (serotype 1 / strain New
            Jersey)
  ORGANISM  Epizootic hemorrhagic disease virus (serotype 1 / strain New
            Jersey)
            Viruses; dsRNA viruses; Reoviridae; Sedoreovirinae; Orbivirus.
REFERENCE   1
  AUTHORS   Anthony,S.J., Maan,N., Maan,S., Sutton,G., Attoui,H. and
            Mertens,P.P.
  TITLE     Genetic and phylogenetic analysis of the core proteins VP1, VP3,
            VP4, VP6 and VP7 of epizootic haemorrhagic disease virus (EHDV)
  JOURNAL   Virus Res. 145 (2), 187-199 (2009)
   PUBMED   19632280
REFERENCE   2  (bases 1 to 3942)
  AUTHORS   Anthony,S.J.
  TITLE     Direct Submission
  JOURNAL   Submitted (30-MAY-2007) Anthony S.J., Department of Arbovirology,
            Institute for Animal Health, Ash Road, Pirbright, Surrey GU24 0NF,
            UK
FEATURES             Location/Qualifiers
     source          1..3942
                     /organism="Epizootic hemorrhagic disease virus (serotype 1
                     / strain New Jersey)"
                     /mol_type="genomic RNA"
                     /strain="New Jersey"
                     /serotype="1"
                     /isolate="USA1955/01"
                     /db_xref="taxon:449133"
                     /segment="1"
                     /country="USA:New Jersey"
                     /collection_date="1955"
     gene            12..3920
                     /gene="VP1"
     CDS             12..3920
                     /gene="VP1"
                     /codon_start=1
                     /product="VP1 protein"
                     /protein_id="CAN99545.1"
                     /db_xref="GI:260161524"
                     /translation="MVAITVQGAELIKRVVERIYPGTSFDLSEGVIEFYKFSDHIRRI
                       ……………………………………………………
                     KRRFKVRAQRFRTHIPRIKVIKKLMDLNRMSVRRLENQFV"
ORIGIN
        1 gttaaaatgc aatggtcgca attaccgtgc aaggtgctga actcattaaa cgagtggttg
       61 aaaggatata cccaggaacg tcatttgatt taagcgaggg cgttattgaa ttttataaat
      121 tttcagacca tataaggcgt attagagaga aacatggggt ggtgtataaa cggaaagcgg
      ……………………………………………………………
     3841 ttccaaggat caaggtaatt aagaaattga tggatttaaa tcgcatgagt gtacgtcgtc
     3901 tagagaacca attcgtttag agtgcacccg cattatactt ac
```

At a preliminary step, GIDL uses an OWL reasoner to make explicit all the admissible relationships among features and to build CLIPS facts representing the knowledge in the Sequence Ontology (see *Appendix*: *OWL reasoner facts*).

1st step: Parser & CLIPS facts

The Parser component produces a set of facts expressed in CLIPS template syntax. Facts generated for data related to the 'gene' and 'CDS' entry sections are boxed.

CLIPS Facts

```
(deffacts initialfacts(
  sequence (parserId "1")(nucleotides "?") )
  (header (parserId 2)(locus "AM744977")(length "3942")(nucleicAcid "RNA")(topology
  "linear")(date "06OCT2009")(definition "Epizootic hemorrhagic disease virus (serotype
  1 / strain New Jersey) segment 1.")(accession "AM744977")(singleAccession
  "AM744977")(gi_number "GI:260161523")(keywords "complete genome segment.")(source
  "Epizootic hemorrhagic disease virus (serotype 1 / strain New Jersey)"))
  (reference (parserId "3") (referenceNumber 2) (authors "Anthony,S.J.")(title "Direct
  Submission")(journal "Submitted (30-MAY-2007) Anthony S.J., Department of
  Arbovirology, Institute for Animal Health, Ash Road, Pirbright, Surrey GU24 0NF, UK")
  )
  (referencePortion (parserId "4")(begin 1)(end 3942)(refId "3") )
  (reference (parserId "5") (referenceNumber "?") (authors "Anthony,S.J., Maan,N.,
  Maan,S., Sutton,G., Attoui,H. and Mertens,P.P.")(title "Genetic and phylogenetic
  analysis of the core proteins VP1, VP3, VP4, VP6 and VP7 of epizootic haemorrhagic
  disease virus (EHDV)")(journal "Virus Res. 145 (2), 187-199 (2009)") )
  (key (parserId "5")(type source)(organism "Epizootic hemorrhagic disease virus
  (serotype 1 / strain New Jersey)" )(mol_type "genomic RNA" )(strain "New Jersey"
  )(serotype "1" )(isolate "USA1955/01" )(db_xref "taxon:449133" )(segment "1" )(country
  "USA:New Jersey" )(collection_date "1955" ))
  (portion (parserId "6")(begin 1)(end 3942)(complement n)(leftComplete y)(rightComplete
  y)(keyId "5") )
```

```
(key (parserId "7")(type gene)(gene "VP1" ))
(portion (parserId "8")(begin 12)(end 3920)(complement n)(leftComplete
y)(rightComplete y)(keyId "7") )
(key (parserId "9")(type CDS)(gene "VP1" )(codon_start "1" )(product "VP1 protein"
)(protein_id "CAN99545.1" )(db_xref "GI:260161524" )(translation
"MVAITVQGAELIKRVVERIYPGTSFDLSEGVIEFYKFSDHIRRIREKHGVVYKRKAEEIERNIKLRKEELFGIPVLRDSTWEEIF
NIDYKDDAVFQVYMNSVLRQEDLEPEEEFLRNYRVHGEHAGLTQFIEQRAKNEMQIYGDIPIKVWAAF
...............................................
...............................................
HTASDLTTVFTLMNIESKVAEELSEYITSEKIRFDALKLLKKGIAGDEFTMSIDVATQAMVDKFIKYPHQLTKTELDAIVLYCSQI
IMLRAACGLPLKKMRLVVLDEAKRRFKVRAQRFRTHIPRIKVIKKLMDLNRMSVRRLENQFV" ))
(portion (parserId "10")(begin 12)(end 3920)(complement n)(leftComplete
y)(rightComplete y)(keyId "9") )
```

```
)
```

2nd step: CLIPS reasoner & quasi-sql facts

The CLIPS reasoner builds quasi-sql facts using the OWL reasoner facts, the previous initial facts describing the GenBank entry and our coded CLIPS rules.

Our coded CLIPS rules allow to:

- transform initial facts into quasi-sql facts (for database insert);
- reconstruct features relationships on the bases of the feature types and of qualifier and location consistency.

Below are reported the CLIPS rules related to “gene” and “CDS” feature keys:

CLIPS Rules

```
(defrule relationshipUsingGene
	(key
		(type ?type_var)
		(parserId ?par_var)
		(gene ?gen_var)
	)
	=>
	(assert (relationshipTracker     gene   ?par_var      ?gen_var)
	)
)


(defrule partConsistence
	(insert
		(tableName sequenceFeaturePortion)
		(foreignCount ?objectCount ?)
		(columnValue ?obj_beg ?obj_end $?)
		)
	(insert
		(tableName sequenceFeaturePortion)
		(count ?portionCount)
		(foreignCount ?subjectCount ?)
		(columnValue ?subj_beg ?subj_end $?)
		)
	(test
		(and
			(>= ?subj_beg ?obj_beg)
			(>= ?obj_end ?subj_end)
			)
		)
	=>
	(assert
		(is-consistent-part-of ?subjectCount ?objectCount ?portionCount)
		)
	)

(defrule objectProperty
	(declare (salience -1))
	(insert
		(tableName sequenceFeature)
		(parserId ?parser_a)
		(count ?a)
```

```
        )
    (insert
        (tableName sequenceFeature_x_ontologyTerm)
        (columnValue ?term_a)
        (foreignCount ?a)
        )
    (insert
        (tableName sequenceFeature)
        (parserId ?parser_b)
        (count ?b)
        )
    (insert
        (tableName sequenceFeature_x_ontologyTerm)
        (columnValue ?term_b)
        (foreignCount ?b)
        )
    (relationship ?term_a ?rel ?term_b)
    (forall
        (insert
                (tableName sequenceFeaturePortion)
                (count ?portionCount)
                (foreignCount ?a ?)
                )
        (is-consistent-part-of ?a ?b ?portionCount)
        )
    (exists
        (relationshipTracker ?type ?parser_a ?tracker)
        (relationshipTracker ?type ?parser_b ?tracker)
        )
    (not (exists
        (relationshipTracker ?type2     ?parser_a ?trackera)
        (relationshipTracker ?type2     ?parser_b ?trackerb)
        (test (neq ?trackera ?trackerb))
        ))
        =>
    (assert
        (insert
            (count ?*count*)
            (tableName featureProperty)
            (columnName termId)
            (columnValue ?rel)
            (foreignColumnName subjectSequenceFeatureId objectSequenceFeatureId)
            (foreignCount ?a ?b)
            )
        )
    (bind ?*count* (+ ?*count* 1))
    )
```

Quasi-sql facts generated by the CLIPS reasoner:

Quasi-SQL Facts

```
(insert (count 38) (tableName sequence) (columnName residues length name description
accession) (columnValue "?" "3942" "AM744977" "Epizootic hemorrhagic disease virus
(serotype 1 / strain New Jersey) segment 1." "AM744977") (foreignColumnName experimentId)
(foreignCount 37) (parserId nil))
(insert (count 21) (tableName reference) (columnName authors title journal) (columnValue
"Anthony,S.J., Maan,N., Maan,S., Sutton,G., Attoui,H. and Mertens,P.P." "Genetic and
phylogenetic analysis of the core proteins VP1, VP3, VP4, VP6 and VP7 of epizootic
haemorrhagic disease virus (EHDV)" "Virus Res. 145 (2), 187-199 (2009)")
(foreignColumnName) (foreignCount) (parserId "5"))
(insert (count 25) (tableName externalDbReference) (columnName accession dateInsert
dbNameId dbnameaccessionconcatenation) (columnValue "AM744977" "06OCT2009" "2"
"nucleotide:AM744977") (foreignColumnName) (foreignCount) (parserId nil))
(insert (count 29) (tableName externalDbReference) (columnName accession dateInsert
dbNameId dbnameaccessionconcatenation) (columnValue "GI:260161523" "06OCT2009" "2"
"nucleotide:GI:260161523") (foreignColumnName) (foreignCount) (parserId nil))
(insert (count 33) (tableName environmentalsampling) (columnName samplingdate country)
(columnValue "1955" "USA:New Jersey") (foreignColumnName) (foreignCount) (parserId nil))
(select (tag taxon) (count 34) (select "taxonid") (from "mblab.referencetaxon") (where
"externalid='449133' and dbnameid=1"))
(insert (count 35) (tableName identification) (columnName) (columnValue)
(foreignColumnName taxonId) (foreignCount 34) (parserId nil))
(insert (count 36) (tableName individual) (columnName) (columnValue) (foreignColumnName
identificationId) (foreignCount 35) (parserId nil))
(insert (count 37) (tableName experiment) (columnName) (columnValue) (foreignColumnName
individualId) (foreignCount 36) (parserId nil))
(insert (count 39) (tableName sequenceAttribute) (columnName termId) (columnValue
"SO:0000987") (foreignColumnName sequenceId) (foreignCount 38) (parserId nil))
(insert (count 40) (tableName sequenceAttribute) (columnName termId) (columnValue
"SO:0000356") (foreignColumnName sequenceId) (foreignCount 38) (parserId nil))
(insert (count 41) (tableName sequenceAttribute) (columnName termId value) (columnValue
"Genbank:keywords" "complete genome segment.") (foreignColumnName sequenceId)
(foreignCount 38) (parserId nil))
(insert (count 42) (tableName externalDbReference_X_Sequence) (columnName) (columnValue)
(foreignColumnName externalDbReferenceId SequenceId) (foreignCount 25 38) (parserId nil))
(insert (count 43) (tableName externalDbReference_X_Sequence) (columnName) (columnValue)
(foreignColumnName externalDbReferenceId SequenceId) (foreignCount 29 38) (parserId nil))
(insert (count 47) (tableName sequenceAttribute) (columnName value termid) (columnValue
"1" "GBQual:segment") (foreignColumnName sequenceId) (foreignCount 38) (parserId nil))
(insert (count 48) (tableName sequenceAttribute) (columnName value termid) (columnValue
"1" "GBQual:serotype") (foreignColumnName sequenceId) (foreignCount 38) (parserId nil))
(insert (count 49) (tableName sequenceAttribute) (columnName value termid) (columnValue
"New Jersey" "GBQual:strain") (foreignColumnName sequenceId) (foreignCount 38) (parserId
nil))
(insert (count 50) (tableName experimentDetails) (columnName samplename) (columnValue
"USA1955/01") (foreignColumnName experimentId) (foreignCount 37) (parserId nil))
```

```
(relationshipTracker gene "9" "VP1")
(relationshipTracker proteinid "9" "CAN99545.1")
(relationshipTracker product "9" "VP1 protein")
(insert (count 0) (tableName sequenceFeature) (columnName) (columnValue)
(foreignColumnName) (foreignCount) (parserId "9"))
(insert (count 1) (tableName sequenceFeatureAnnotation) (columnName value termid)
(columnValue "1" "GBQual:codon_start") (foreignColumnName sequenceFeatureId)
(foreignCount 0) (parserId nil))
```

```
(insert (count 2) (tableName sequenceFeatureAnnotation) (columnName value termid)
(columnValue "GI:260161524" "GBQual:db_xref") (foreignColumnName sequenceFeatureId)
(foreignCount 0) (parserId nil))
(insert (count 3) (tableName sequenceFeatureAnnotation) (columnName value termid)
(columnValue "VP1" "GBQual:gene") (foreignColumnName sequenceFeatureId) (foreignCount 0)
(parserId nil))
(insert (count 4) (tableName sequenceFeatureAnnotation) (columnName value termid)
(columnValue "VP1 protein" "GBQual:product") (foreignColumnName sequenceFeatureId)
(foreignCount 0) (parserId nil))
(insert (count 5) (tableName sequenceFeatureAnnotation) (columnName value termid)
(columnValue "CAN99545.1" "GBQual:protein_id") (foreignColumnName sequenceFeatureId)
(foreignCount 0) (parserId nil))
(insert (count 6) (tableName sequenceFeature_x_ontologyTerm) (columnName termId)
(columnValue "SO:0000316") (foreignColumnName sequenceFeatureId) (foreignCount 0)
(parserId nil))
(relationshipTracker gene "7" "VP1")
(insert (count 7) (tableName sequenceFeature) (columnName) (columnValue)
(foreignColumnName) (foreignCount) (parserId "7"))
(insert (count 8) (tableName sequenceFeatureAnnotation) (columnName value termid)
(columnValue "VP1" "GBQual:gene") (foreignColumnName sequenceFeatureId) (foreignCount 7)
(parserId nil))
(insert (count 9) (tableName sequenceFeature_x_ontologyTerm) (columnName termId)
(columnValue "SO:0000704") (foreignColumnName sequenceFeatureId) (foreignCount 7)
(parserId nil))
```

```
(sourceLength "5" 3941)
(insert (count 10) (tableName sequenceFeature) (columnName) (columnValue)
(foreignColumnName) (foreignCount) (parserId "5"))
(insert (count 11) (tableName sequenceFeatureAnnotation) (columnName value termid)
(columnValue "1955" "GBQual:collection_date") (foreignColumnName sequenceFeatureId)
(foreignCount 10) (parserId nil))
(insert (count 12) (tableName sequenceFeatureAnnotation) (columnName value termid)
(columnValue "USA:New Jersey" "GBQual:country") (foreignColumnName sequenceFeatureId)
(foreignCount 10) (parserId nil))
(insert (count 13) (tableName sequenceFeatureAnnotation) (columnName value termid)
(columnValue "taxon:449133" "GBQual:db_xref") (foreignColumnName sequenceFeatureId)
(foreignCount 10) (parserId nil))
(insert (count 14) (tableName sequenceFeatureAnnotation) (columnName value termid)
(columnValue "USA1955/01" "GBQual:isolate") (foreignColumnName sequenceFeatureId)
(foreignCount 10) (parserId nil))
(insert (count 15) (tableName sequenceFeatureAnnotation) (columnName value termid)
(columnValue "genomic RNA" "GBQual:mol_type") (foreignColumnName sequenceFeatureId)
(foreignCount 10) (parserId nil))
(insert (count 16) (tableName sequenceFeatureAnnotation) (columnName value termid)
(columnValue "Epizootic hemorrhagic disease virus (serotype 1 / strain New Jersey)"
"GBQual:organism") (foreignColumnName sequenceFeatureId) (foreignCount 10) (parserId
nil))
(insert (count 17) (tableName sequenceFeatureAnnotation) (columnName value termid)
(columnValue "1" "GBQual:segment") (foreignColumnName sequenceFeatureId) (foreignCount
10) (parserId nil))
(insert (count 18) (tableName sequenceFeatureAnnotation) (columnName value termid)
(columnValue "1" "GBQual:serotype") (foreignColumnName sequenceFeatureId) (foreignCount
10) (parserId nil))
(insert (count 19) (tableName sequenceFeatureAnnotation) (columnName value termid)
(columnValue "New Jersey" "GBQual:strain") (foreignColumnName sequenceFeatureId)
(foreignCount 10) (parserId nil))
(insert (count 20) (tableName sequenceFeature_x_ontologyTerm) (columnName termId)
(columnValue "SO:2000061") (foreignColumnName sequenceFeatureId) (foreignCount 10)
(parserId nil))
```

```
(insert (count 22) (tableName sequenceFeatureEvidence) (columnName) (columnValue)
(foreignColumnName referenceId sequenceFeatureId) (foreignCount 21 0) (parserId nil))
(insert (count 23) (tableName sequenceFeatureEvidence) (columnName) (columnValue)
(foreignColumnName referenceId sequenceFeatureId) (foreignCount 21 7) (parserId nil))
(insert (count 24) (tableName sequenceFeatureEvidence) (columnName) (columnValue)
(foreignColumnName referenceId sequenceFeatureId) (foreignCount 21 10) (parserId nil))
(insert (count 26) (tableName externalDbReference_X_SequenceFeature) (columnName)
(columnValue) (foreignColumnName sequenceFeatureId externalDbReferenceId) (foreignCount 0
25) (parserId nil))
(insert (count 27) (tableName externalDbReference_X_SequenceFeature) (columnName)
(columnValue) (foreignColumnName sequenceFeatureId externalDbReferenceId) (foreignCount 7
25) (parserId nil))
(insert (count 28) (tableName externalDbReference_X_SequenceFeature) (columnName)
(columnValue) (foreignColumnName sequenceFeatureId externalDbReferenceId) (foreignCount
10 25) (parserId nil))
(insert (count 30) (tableName externalDbReference_X_SequenceFeature) (columnName)
(columnValue) (foreignColumnName sequenceFeatureId externalDbReferenceId) (foreignCount 0
29) (parserId nil))
(insert (count 31) (tableName externalDbReference_X_SequenceFeature) (columnName)
(columnValue) (foreignColumnName sequenceFeatureId externalDbReferenceId) (foreignCount 7
29) (parserId nil))
(insert (count 32) (tableName externalDbReference_X_SequenceFeature) (columnName)
(columnValue) (foreignColumnName sequenceFeatureId externalDbReferenceId) (foreignCount
10 29) (parserId nil))
(mainSource "5")
```

```
(insert (count 44) (tableName sequenceFeaturePortion) (columnName begin end Complement
leftComplete rightComplete) (columnValue 12 3920 n y y) (foreignColumnName
sequenceFeatureId sequenceId) (foreignCount 0 38) (parserId "10"))
(is-consistent-part-of 0 0 44)
(insert (count 45) (tableName sequenceFeaturePortion) (columnName begin end Complement
leftComplete rightComplete) (columnValue 12 3920 n y y) (foreignColumnName
sequenceFeatureId sequenceId) (foreignCount 7 38) (parserId "8"))
(is-consistent-part-of 7 7 45)
(is-consistent-part-of 0 7 44)
(is-consistent-part-of 7 0 45)
```

```
(insert (count 46) (tableName sequenceFeaturePortion) (columnName begin end Complement
leftComplete rightComplete) (columnValue 1 3942 n y y) (foreignColumnName
sequenceFeatureId sequenceId) (foreignCount 10 38) (parserId "6"))
(is-consistent-part-of 10 10 46)
(is-consistent-part-of 7 10 45)
(is-consistent-part-of 0 10 44)
```

```
(insert (count 51) (tableName featureProperty) (columnName termId) (columnValue
"part_of") (foreignColumnName subjectSequenceFeatureId objectSequenceFeatureId)
(foreignCount 0 7) (parserId nil))
```

3rd step: DBFiller & SQL statements

The DBFiller component of GIDL translates the quasi-sql facts, generated by the CLIPS reasoner, into real SQL statements that are used to populate the Target Database.

Below are reported the SQL statements generated for the GenBank entry AM744977. The SQL statements related to the "gene" and "CDS" feature keys are boxed.

SQL Statements

```
INSERT INTO MBLAB.sequence (residues,length,name,description,accession,experimentId)
VALUES (?,3942,'AM744977','Epizootic hemorrhagic disease virus (serotype 1 / strain New
Jersey) segment 1.','AM744977',122396)
INSERT INTO MBLAB.reference (authors,title,journal) VALUES ('Anthony,S.J., Maan,N.,
Maan,S., Sutton,G., Attoui,H. and Mertens,P.P.','Genetic and phylogenetic analysis of the
core proteins VP1, VP3, VP4, VP6 and VP7 of epizootic haemorrhagic disease virus
(EHDV)','Virus Res. 145 (2), 187-199 (2009)')
INSERT INTO MBLAB.externalDbReference
(accession,dateInsert,dbNameId,dbnameaccessionconcatenation) VALUES
('AM744977','06.10.2009',2,'nucleotide:AM744977')
INSERT INTO MBLAB.externalDbReference
(accession,dateInsert,dbNameId,dbnameaccessionconcatenation) VALUES
('GI:260161523','06.10.2009',2,'nucleotide:GI:260161523')
INSERT INTO MBLAB.environmentalsampling (samplingdate,country) VALUES
('01.01.1955','USA:New Jersey')
select taxonid from mblab.referencetaxon where externalid='449133' and dbnameid=1
INSERT INTO MBLAB.identification (taxonId) VALUES (449133)
INSERT INTO MBLAB.individual (identificationId) VALUES (122396)
INSERT INTO MBLAB.experiment (individualId) VALUES (122396)
INSERT INTO MBLAB.sequenceAttribute (termId,sequenceId) VALUES ('SO:0000987',122396)
INSERT INTO MBLAB.sequenceAttribute (termId,sequenceId) VALUES ('SO:0000356',122396)
INSERT INTO MBLAB.sequenceAttribute (termId,value,sequenceId) VALUES
('Genbank:keywords','complete genome segment.',122396)
```

```
INSERT INTO MBLAB.sequenceFeature (SEQUENCEFEATUREID) VALUES (DEFAULT)
INSERT INTO MBLAB.sequenceFeatureAnnotation (value,termid,sequenceFeatureId) VALUES
('1','GBQual:codon_start',466428)
INSERT INTO MBLAB.sequenceFeatureAnnotation (value,termid,sequenceFeatureId) VALUES
('GI:260161524','GBQual:db_xref',466428)
INSERT INTO MBLAB.sequenceFeatureAnnotation (value,termid,sequenceFeatureId) VALUES
('VP1','GBQual:gene',466428)
INSERT INTO MBLAB.sequenceFeatureAnnotation (value,termid,sequenceFeatureId) VALUES ('VP1
protein','GBQual:product',466428)
INSERT INTO MBLAB.sequenceFeatureAnnotation (value,termid,sequenceFeatureId) VALUES
('CAN99545.1','GBQual:protein_id',466428)
INSERT INTO MBLAB.sequenceFeature_x_ontologyTerm (termId,sequenceFeatureId) VALUES
('SO:0000316',466428)
INSERT INTO MBLAB.sequenceFeature (SEQUENCEFEATUREID) VALUES (DEFAULT)
INSERT INTO MBLAB.sequenceFeatureAnnotation (value,termid,sequenceFeatureId) VALUES
('VP1','GBQual:gene',466429)
INSERT INTO MBLAB.sequenceFeature_x_ontologyTerm (termId,sequenceFeatureId) VALUES
('SO:0000704',466429)
```

```
INSERT INTO MBLAB.sequenceFeature (SEQUENCEFEATUREID) VALUES (DEFAULT)
INSERT INTO MBLAB.sequenceFeatureAnnotation (value,termid,sequenceFeatureId) VALUES
('1955','GBQual:collection_date',466430)
INSERT INTO MBLAB.sequenceFeatureAnnotation (value,termid,sequenceFeatureId) VALUES
('USA:New Jersey','GBQual:country',466430)
INSERT INTO MBLAB.sequenceFeatureAnnotation (value,termid,sequenceFeatureId) VALUES
('taxon:449133','GBQual:db_xref',466430)
```

```
INSERT INTO MBLAB.sequenceFeatureAnnotation (value,termid,sequenceFeatureId) VALUES ('USA1955/01','GBQual:isolate',466430)
INSERT INTO MBLAB.sequenceFeatureAnnotation (value,termid,sequenceFeatureId) VALUES ('genomic RNA','GBQual:mol_type',466430)
INSERT INTO MBLAB.sequenceFeatureAnnotation (value,termid,sequenceFeatureId) VALUES ('Epizootic hemorrhagic disease virus (serotype 1 / strain New Jersey)','GBQual:organism',466430)
INSERT INTO MBLAB.sequenceFeatureAnnotation (value,termid,sequenceFeatureId) VALUES ('1','GBQual:segment',466430)
INSERT INTO MBLAB.sequenceFeatureAnnotation (value,termid,sequenceFeatureId) VALUES ('1','GBQual:serotype',466430)
INSERT INTO MBLAB.sequenceFeatureAnnotation (value,termid,sequenceFeatureId) VALUES ('New Jersey','GBQual:strain',466430)
INSERT INTO MBLAB.sequenceFeature_x_ontologyTerm (termId,sequenceFeatureId) VALUES ('SO:2000061',466430)
```

INSERT INTO MBLAB.sequenceFeatureEvidence (referenceId,sequenceFeatureId) VALUES (119974,466428)
INSERT INTO MBLAB.sequenceFeatureEvidence (referenceId,sequenceFeatureId) VALUES (119974,466429)

```
INSERT INTO MBLAB.sequenceFeatureEvidence (referenceId,sequenceFeatureId) VALUES (119974,466430)
```

INSERT INTO MBLAB.externalDbReference_X_SequenceFeature (sequenceFeatureId,externalDbReferenceId) VALUES (466428,244973)
INSERT INTO MBLAB.externalDbReference_X_SequenceFeature (sequenceFeatureId,externalDbReferenceId) VALUES (466429,244973)
INSERT INTO MBLAB.externalDbReference_X_SequenceFeature (sequenceFeatureId,externalDbReferenceId) VALUES (466430,244973)
INSERT INTO MBLAB.externalDbReference_X_SequenceFeature (sequenceFeatureId,externalDbReferenceId) VALUES (466428,244974)
INSERT INTO MBLAB.externalDbReference_X_SequenceFeature (sequenceFeatureId,externalDbReferenceId) VALUES (466429,244974)

```
INSERT INTO MBLAB.externalDbReference_X_SequenceFeature (sequenceFeatureId,externalDbReferenceId) VALUES (466430,244974)
INSERT INTO MBLAB.externalDbReference_X_Sequence (externalDbReferenceId,SequenceId) VALUES (244973,122396)
INSERT INTO MBLAB.externalDbReference_X_Sequence (externalDbReferenceId,SequenceId) VALUES (244974,122396)
```

INSERT INTO MBLAB.sequenceFeaturePortion (begin,end,Complement,leftComplete,rightComplete,sequenceFeatureId,sequenceId) VALUES (12,3920,'n','y','y',466428,122396)
INSERT INTO MBLAB.sequenceFeaturePortion (begin,end,Complement,leftComplete,rightComplete,sequenceFeatureId,sequenceId) VALUES (12,3920,'n','y','y',466429,122396)

```
INSERT INTO MBLAB.sequenceFeaturePortion (begin,end,Complement,leftComplete,rightComplete,sequenceFeatureId,sequenceId) VALUES (1,3942,'n','y','y',466430,122396)
INSERT INTO MBLAB.sequenceAttribute (value,termid,sequenceId) VALUES ('1','GBQual:segment',122396)
INSERT INTO MBLAB.sequenceAttribute (value,termid,sequenceId) VALUES ('1','GBQual:serotype',122396)
INSERT INTO MBLAB.sequenceAttribute (value,termid,sequenceId) VALUES ('New Jersey','GBQual:strain',122396)
INSERT INTO MBLAB.experimentDetails (samplename,experimentId) VALUES ('USA1955/01',122396)
```

INSERT INTO MBLAB.featureProperty (termId,subjectSequenceFeatureId,objectSequenceFeatureId) VALUES ('part_of',466428,466429)

Appendix: OWL reasoner facts

This section shows a portion of the CLIPS facts generated by the OWL reasoner at a preliminary stage, una tantum, in the setup phase. The facts used for the processing of the GenBank entry AM744977 are highlighted.

```
(topology "linear" "SO:0000987")
(topology "circular" "SO:0000988")
(nucleicAcid "DNA" "SO:0000352")
(nucleicAcid "RNA" "SO:0000356")
(nucleicAcid "mRNA" "SO:0000356")
(nucleicAcid "tRNA" "SO:0000356")
(nucleicAcid "rRNA" "SO:0000356")
(nucleicAcid "snRNA" "SO:0000356")
(nucleicAcid "uRNA" "SO:0000356")
(nucleicAcid "snoRNA" "SO:0000356")
(sequenceOntology -10_signal "SO:0000175")
(sequenceOntology -35_signal "SO:0000176")
(sequenceOntology 3'UTR "SO:0000205")
(sequenceOntology 3'clip "SO:0000557")
(sequenceOntology 5'UTR "SO:0000204")
(sequenceOntology 5'clip "SO:0000555")
(sequenceOntology CAAT_signal "SO:0000172")
(sequenceOntology CDS "SO:0000316")
(sequenceOntology D-loop "SO:0000297")
(sequenceOntology D_segment "SO:0000458")
(sequenceOntology GC_signal "SO:0000173")
(sequenceOntology LTR "SO:0000286")
(sequenceOntology RBS "SO:0000139")
(sequenceOntology STS "SO:0000331")
(sequenceOntology TATA_signal "SO:0000174")
(sequenceOntology attenuator "SO:0000140")
(sequenceOntology enhancer "SO:0000165")
(sequenceOntology exon "SO:0000147")
(sequenceOntology gap "SO:0000730")
(sequenceOntology gene "SO:0000704")
(sequenceOntology iDNA "SO:0000723")
(sequenceOntology intron "SO:0000188")
(sequenceOntology mRNA "SO:0000234")
(sequenceOntology mat_peptide "SO:0000419")
(sequenceOntology misc_RNA "SO:0000673")
(sequenceOntology misc_binding "SO:0000409")
(sequenceOntology misc_difference "SO:0000413")
(sequenceOntology misc_feature "SO:0000001")
(sequenceOntology misc_recomb "SO:0000298")
(sequenceOntology misc_signal "SO:0005836")
(sequenceOntology misc_structure "SO:0000002")
(sequenceOntology modified_base "SO:0000305")
(sequenceOntology ncRNA "SO:0000655")
(sequenceOntology operon "SO:0000178")
(sequenceOntology oriT "SO:0000724")
(sequenceOntology polyA_signal "SO:0000551")
(sequenceOntology polyA_site "SO:0000553")
(sequenceOntology precursor_RNA "SO:0000185")
(sequenceOntology prim_transcript "SO:0000185")
(sequenceOntology primer_bind "SO:0005850")
(sequenceOntology promoter "SO:0000167")
(sequenceOntology protein_bind "SO:0000410")
(sequenceOntology rRNA "SO:0000252")
(sequenceOntology rep_origin "SO:0000296")
(sequenceOntology repeat_region "SO:0000657")
(sequenceOntology repeat_unit "SO:0000726")
(sequenceOntology satellite "SO:0000005")
(sequenceOntology scRNA "SO:0000013")
(sequenceOntology sig_peptide "SO:0000418")
```

```
(sequenceOntology snRNA "SO:0000274")
(sequenceOntology snoRNA "SO:0000275")
(sequenceOntology source "SO:2000061")
(sequenceOntology stem_loop "SO:0000313")
(sequenceOntology tRNA "SO:0000253")
(sequenceOntology terminator "SO:0000141")
(sequenceOntology transit_peptide "SO:0000725")
(sequenceOntology variation "SO:0001060")
(sequenceOntology conflict "SO:0000702")
(relationship "SO:0000141" "member_of" "SO:0000001")
(relationship "SO:0000173" "part_of" "SO:0005836")
(relationship "SO:0000286" "part_of" "SO:0000001")
(relationship "SO:0000730" "part_of" "SO:0000001")
(relationship "SO:0000204" "part_of" "SO:0000001")
(relationship "SO:0000205" "part_of" "SO:0000704")
(relationship "SO:0000555" "part_of" "SO:0000185")
(relationship "SO:0000275" "derives_from" "SO:0000001")
(relationship "SO:0000140" "part_of" "SO:0000001")
(relationship "SO:0000139" "part_of" "SO:0000234")
(relationship "SO:0000253" "derives_from" "SO:0000185")
(relationship "SO:0000185" "part_of" "SO:0000001")
(relationship "SO:0000316" "part_of" "SO:0000001")
(relationship "SO:0000316" "part_of" "SO:0000704")
(relationship "SO:0000316" "part_of" "SO:0000234")
(relationship "SO:0000316" "part_of" "SO:0000673")
(relationship "SO:0000724" "part_of" "SO:0000001")
(relationship "SO:0000205" "part_of" "SO:0000001")
(relationship "SO:0000275" "derives_from" "SO:0000673")
(relationship "SO:0000553" "part_of" "SO:0000704")
(relationship "SO:0000141" "part_of" "SO:0000704")
(relationship "SO:0000185" "member_of" "SO:0000001")
```
……………………………………
